# Visualizing the invisible: inner plexiform layer stratification with conventional spectral-domain optical coherence tomography

**DOI:** 10.1186/s40942-025-00692-3

**Published:** 2025-06-15

**Authors:** Ricardo Luz Leitão Guerra, Luiz Roisman, Jay S. Duker, Giuseppe Querques, Luiz Filipe Adami Lucatto, Emmerson Badaró, Gabriel Castilho S. Barbosa, Eduardo Amorim Novais

**Affiliations:** 1 Retina Department, Leitão Guerra - Oftalmologia, Salvador, Brazil; 2 Orbit Ophthalmo Learning, Salvador, Brazil; 3 Department of Ophthalmology, Federal University of São Paulo, São Paulo, Brazil; 4 New England Eye Center, Tufts Medical Center, Boston, MA USA; 5 Tufts University School of Medicine, Boston, MA USA; 6 School of Medicine, Vita-Salute San Raffaele University, Milan, Italy; 7 Division of head and neck, Medical Retina & Imaging Unit, IRCCS San Raffaele Scientific Institute, Milan, Italy; 8 Department of Ophthalmology, University of São Paulo, São Paulo, Brazil; 9 Cole Eye Institute, Cleveland Clinic, Cleveland, OH USA

**Keywords:** Inner plexiform layer, Optical coherence tomography, Spectral-domain, Retinal imaging

## Abstract

**Background:**

The inner plexiform layer (IPL) of the retina plays a key role in visual processing, consisting of five stratified sub-bands (S1-S5) that segregate ON and OFF visual pathways. Until now, resolving these IPL sub-layers was only possible with experimental high-resolution (HR-OCT) or visible-light OCT (VIS-OCT), which remain inaccessible for clinical use. This study provides the first demonstration that IPL stratification can be visualized using commercially available spectral-domain OCT (SD-OCT) with optimized imaging and grayscale inversion.

**Methods:**

This retrospective, cross-sectional image analysis study included three healthy individuals who underwent macular OCT imaging. Two subjects were imaged with SD-OCT devices (Nidek RS3000 Advance and Zeiss Cirrus 6000), while one subject was imaged with a swept-source OCT (SS-OCT) device (Topcon Triton DRI). High-density B-scans (1024 A-scans per B-scan) with 120 repetitions for noise reduction were analyzed in both standard and inverted grayscale display modes. The impact of scan size (12 mm, 6 mm, and 3 mm) on IPL visualization was also evaluated.

**Results:**

In conventional grayscale, IPL stratification was indistinct. However, inverted grayscale revealed five IPL sub-bands in all cases, particularly in the parafoveal region where the IPL is thicker. Hyperreflective dots near IPL-1, likely representing the superficial capillary plexus, were also identified. The 3-mm scan protocol provided superior sub-layer differentiation compared to 12-mm scans. However, SS-OCT images did not allow for the distinction of the five IPL strata.

**Conclusions:**

This study challenges the belief that IPL stratification cannot be identified with conventional SD-OCT. By refining imaging parameters and using grayscale inversion, this approach enhances retinal circuit analysis with standard technology. While SD-OCT enables detailed IPL visualization under specific conditions, SS-OCT does not appear to be well-suited for this purpose. These findings redefine SD-OCT’s diagnostic capabilities, opening avenues for research in ophthalmology and neurodegenerative disease monitoring. Further studies should establish best practices and expand clinical applications for this novel methodology.

## Background

The inner plexiform layer (IPL) of the retina is a synaptic network where bipolar, amacrine, and ganglion cells process visual information, supported by Müller cell processes that stabilize neuronal arrangement. This intricate layer exhibits a distinct stratification pattern, with five sub-bands or stratas (S1-S5) corresponding to the segregation of ON and OFF signaling pathways [1]. These pathways represent fundamental mechanisms for encoding light increments and decrements, respectively, and their anatomical segregation within the IPL highlights the parallel processing nature of retinal circuitry [2].

Conventional spectral-domain optical coherence tomography (SD-OCT) is routinely used to image retinal structures. However, its limited axial resolution (5–7 μm) typically prevents the visualization of individual IPL sub-bands [2]. Recent studies using experimental high-resolution OCT (HR-OCT) systems have demonstrated the capability to resolve these five strata in vivo. HR-OCT achieves finer axial resolution (less than 3 μm) by employing a broader bandwidth light source and shorter central wavelengths [2, 3].

Another experimental approach, visible light OCT (VIS-OCT), utilizes even shorter wavelengths in the visible spectrum to achieve an axial resolution of 1.0 μm. This five-fold improvement over conventional OCT has enabled the visualization of subtle variations in reflectivity within the IPL, possibly corresponding to different cell types and synaptic structures [2, 3]. Despite their remarkable capabilities, both HR-OCT and VIS-OCT remain experimental and are not commercially available. This limited accessibility restricts the widespread application of these technologies for detailed IPL analysis in clinical settings [2, 3].

The cross-sectional images obtained by SD- and swept-source (SS) OCT devices are typically displayed in a standard grayscale format. In this format, hyper-reflective structures (which return a stronger signal) appear white or in lighter shades, while hypo-reflective structures are displayed in darker tones. However, it is possible to invert this grayscale (inverted grayscale or negative display image), causing hyper-reflective regions to appear black and hypo-reflective regions to appear white. This presentation does not alter the underlying information; it simply “inverts” the image, transforming light pixels into dark pixels and vice versa.

This study aims to evaluate whether the five distinct sub-bands of the IPL can be visualized using commercially available SD- and SS-OCT devices by optimizing imaging parameters and utilizing inverted grayscale format. Unlike experimental HR- and VIS-OCT systems that require hardware modifications to achieve submicron axial resolution, our approach relies solely on standard clinical devices, without post-processing or specialized software. By doing so, we aim to assess whether meaningful visualization of IPL sublayering is achievable in real-world settings, acknowledging the inherent limitations of SD-OCT resolution.

## Methods

This study was designed as a cross-sectional imaging analysis using a convenience sample of three healthy individuals. The primary objective was to evaluate whether IPL stratification could be visualized using commercially available SD- and SS-OCT with optimized imaging parameters and inverted grayscale format.

Macular OCT imaging was performed using three devices: Nidek RS3000 Advance (Nidek Co., Ltd., Japan) and Zeiss Cirrus 6000 (Carl Zeiss Meditec, Germany) for SD-OCT, and Topcon Triton DRI (Topcon Corporation, Japan) for SS-OCT. The available scans included horizontal and vertical B-scans with scan lengths of 12 mm, 6 mm, and 3 mm. All images had been acquired using device-specific settings, following a high-density protocol of 1024 A-scans per B-scan. Although reproducibility was not formally tested, scans were acquired using high-density protocols with 120 repetitions and active eye tracking to ensure precise alignment and minimize motion artifacts.

B-scan images were analyzed using the proprietary software of each device, which allows toggling between standard grayscale and inverted grayscale visualizations. The grayscale inversion function was applied directly within the device, and no contrast enhancement, magnification adjustment, or post-processing was performed. This was done to preserve the original image characteristics and simulate real-world clinical visualization. This ensured that the findings reflected the imaging capabilities of commercially available OCT devices. The analysis focused on the visibility of IPL stratification in standard and inverted grayscale display modes, as well as the impact of scan length on the differentiation of IPL sub-bands.

Two independent ophthalmologists (RLLG and LR) evaluated the images for IPL sub-layer differentiation. In cases of disagreement, a third reviewer (EAN) adjudicated the findings to reach a consensus. The evaluation specifically assessed whether IPL stratification was distinguishable and whether the visualization was affected by scan length or grayscale inversion.

## Results

This study describes three normal subjects (healthy emmetropic individuals) who underwent macular OCT imaging. Two subjects were imaged with SD-OCT using the, while one subject was imaged with SS-OCT. The participants were asymptomatic, with no comorbidities or significant medical or ophthalmologic history. They also presented with uncorrected visual acuity of 20/20, and their comprehensive ophthalmologic examinations revealed no significant findings.

**Fig. 1:**
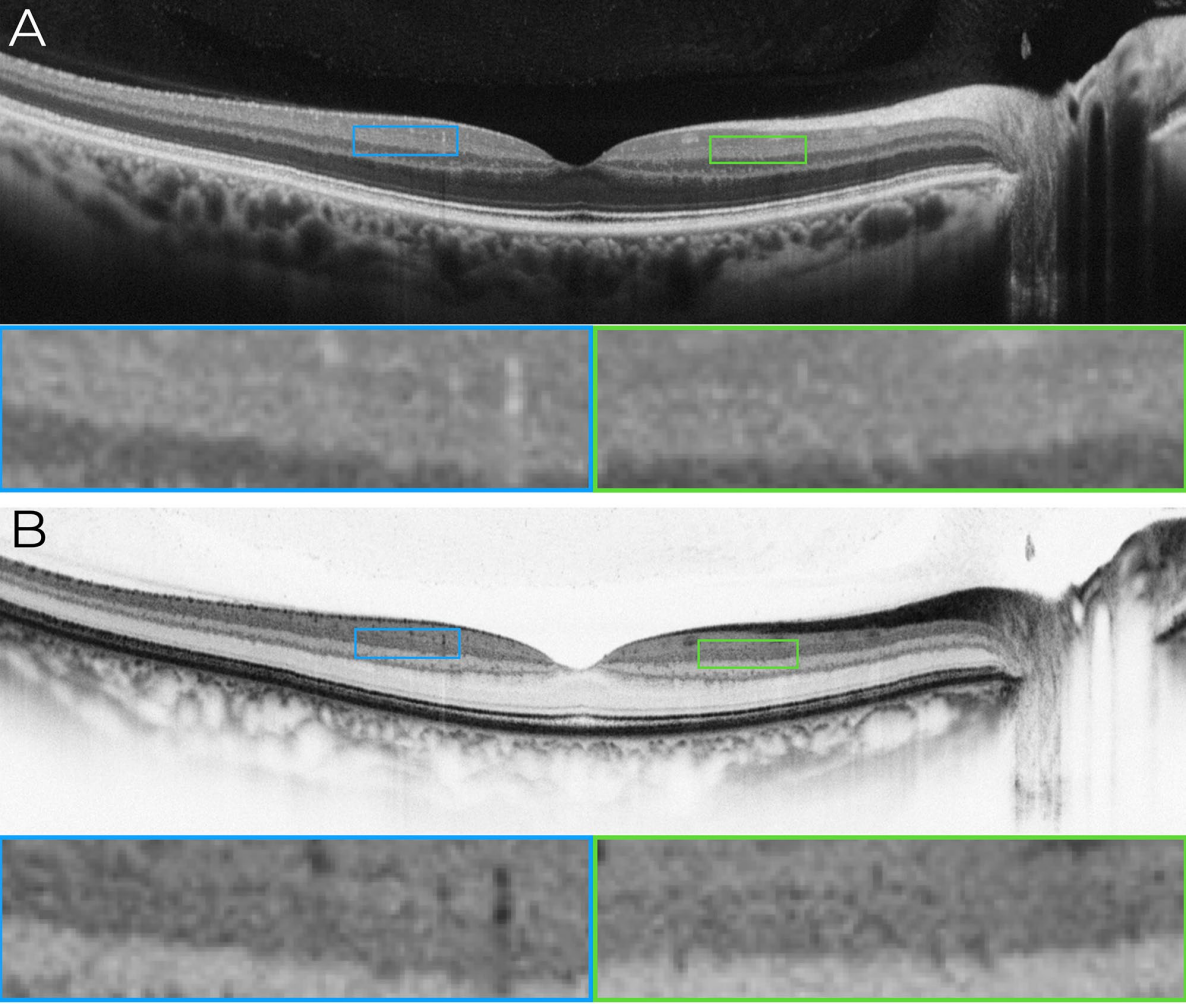
Horizontal 12-mm macular OCT B-scan illustrating the inner plexiform layer (IPL) in standard and inverted grayscale modes. (**A**) Standard grayscale OCT image acquired using a horizontal 12-mm scan with the Nidek RS3000 Advance device. The scan parameters included ultra-fine resolution, active eye tracking, 1024 A-scans per B-scan, and 120 repetitions of B-scans for digital image enhancement (denoising). In this standard grayscale mode, the differentiation of the IPL into its five distinct sub-bands (IPL 1– IPL 5) is not evident, as the hyper-reflective and hypo-reflective layers within the IPL remain indistinct. Magnified views of the parafoveal region (blue and green boxes) highlight the absence of a clear stratification. (**B**) Inverted grayscale OCT image of the same horizontal scan. By reversing the grayscale polarity, hyper-reflective regions appear black, and hypo-reflective regions appear white, enhancing the visualization of the IPL’s sub-bands. Partial delineation of hyper-reflective lines corresponding to the IPL strata becomes discernible, as indicated in the magnified parafoveal regions (blue and green boxes). These findings illustrate that inverted grayscale enhances contrast within the IPL, facilitating the identification of its multilaminar architecture

**Fig. 2:**
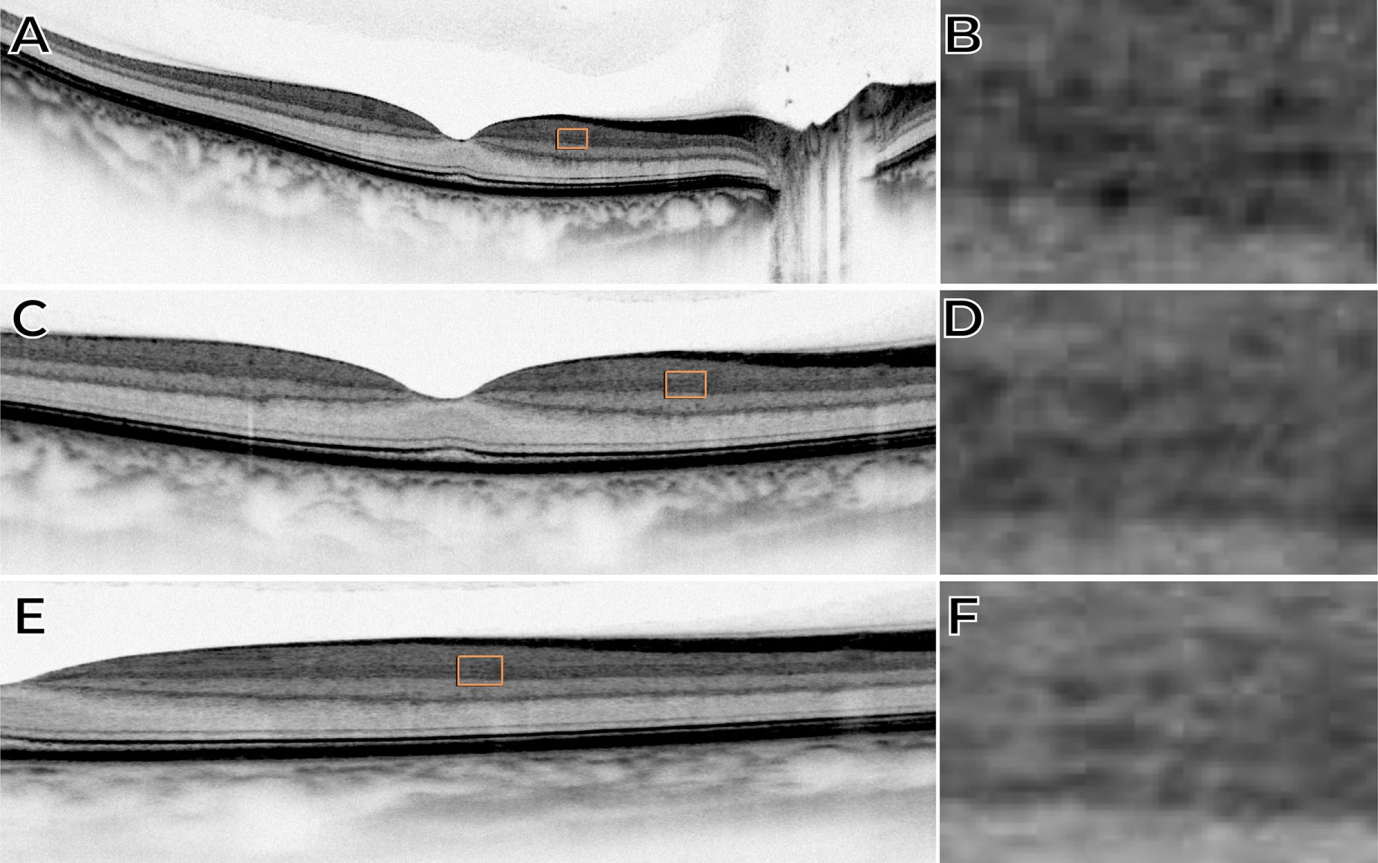
Horizontal macular OCT B-scan acquired with the Zeiss Cirrus 6000 device illustrating the inner plexiform layer (IPL) in inverted grayscale mode using 12-mm, 6-mm, and 3-mm scan lengths. (**A**) 12-mm scan with a highlighted area shown at higher magnification in (**B**). (**C**) 6-mm scan with a highlighted area shown in (**D**). (**E**) 3-mm scan with a highlighted area shown in (**F**). In all scans, the differentiation of the IPL into its five distinct sub-bands (IPL 1– IPL 5) is visible, with smaller scan lengths providing more evident stratification

**Fig. 3:**
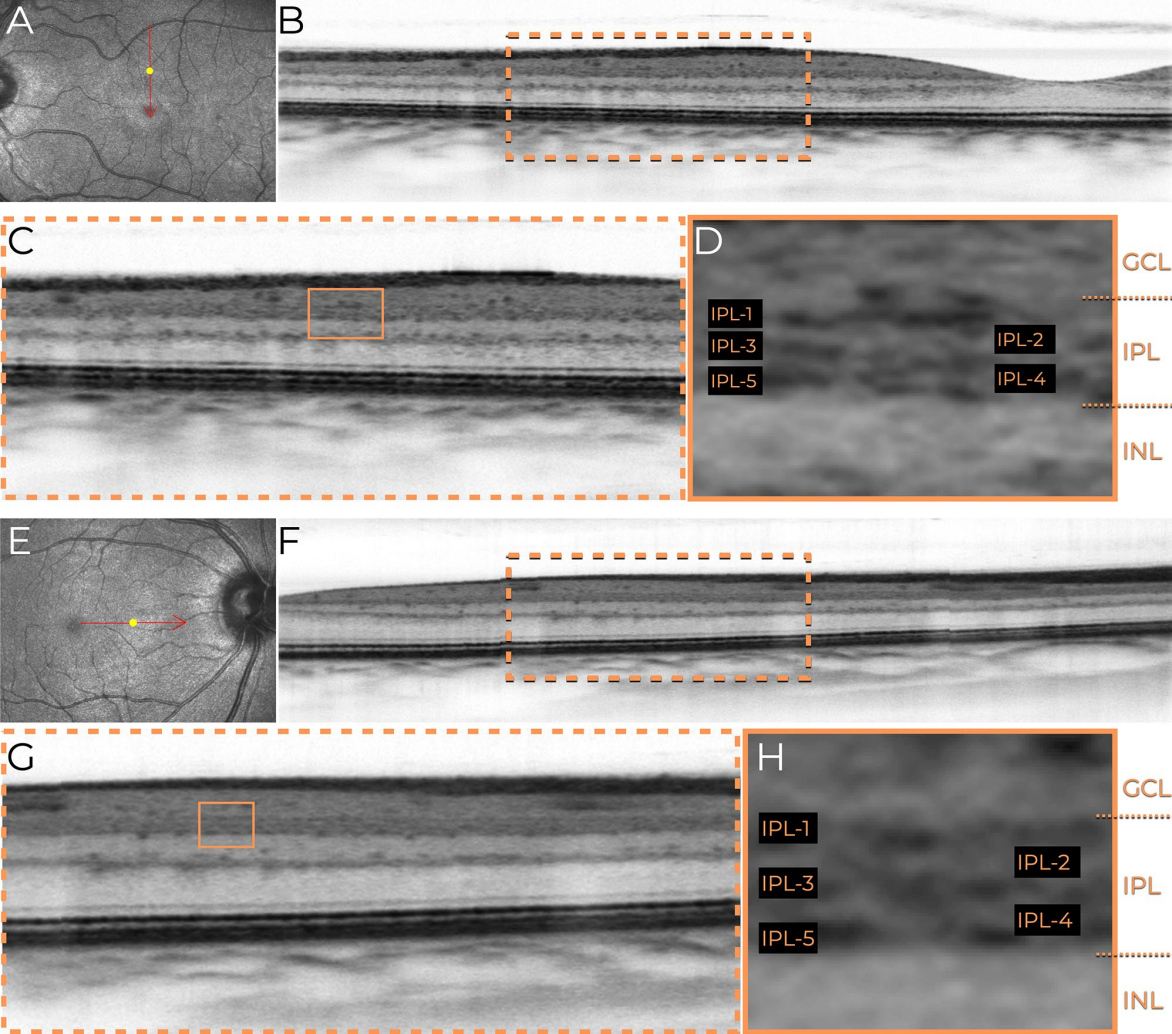
Macular OCT imaging using horizontal and vertical 3-mm scans to visualize the inner plexiform layer (IPL) in high detail. (**A**) Infrared fundus image illustrating the location of the vertical scan (red arrow indicating the scan direction). (**B**) Horizontal 3-mm macular B-scan obtained using the Nidek RS3000 Advance device. The scan parameters included ultra-fine resolution, active eye tracking, 1024 A-scans per B-scan, and 120 repetitions of B-scans for digital image enhancement (denoising). The orange dashed rectangle highlights the parafoveal area, shown in greater detail in panel C. (**C**) Magnified view of the parafoveal area (orange dashed rectangle in panel B), allowing the identification of five distinct sub-bands (IPL-1 to IPL-5) that represent the IPL’s stratification. (**D**) Detailed annotation of the IPL sub-bands (IPL-1 to IPL-5) within the magnified region (orange solid rectangle in panel **C**). Adjacent retinal layers, including the ganglion cell layer (GCL) and inner nuclear layer (INL), are labeled to provide anatomical context. (**E**) Infrared fundus image illustrating the location of the horizontal scan (red arrow indicating the scan direction). (**F**) Horizontal 3-mm macular B-scan using the same parameters as the vertical scan. The orange dashed rectangle highlights the parafoveal area, shown in greater detail in panel G. (**G**) Magnified view of the parafoveal area (orange dashed rectangle in panel F), allowing the identification of five distinct sub-bands (IPL-1 to IPL-5) that represent the IPL’s stratification. (**H**) Detailed annotation of the IPL sub-bands (IPL-1 to IPL-5) within the magnified region (orange solid rectangle in panel G). Adjacent retinal layers, including the GCL and INL, are labeled to provide anatomical context

Case 1 involved a 29-year-old woman who underwent macular OCT imaging with horizontal 12-mm, 6-mm, and 3-mm scans (Figs. 1 and 2). Case 2 involved a 39-year-old man who underwent macular OCT imaging with horizontal and vertical 3-mm scans (Fig. 3). Case 3 involved a 39-year-old man who underwent macular OCT imaging with a 6.0-mm horizontal scan.

**Fig. 4:**
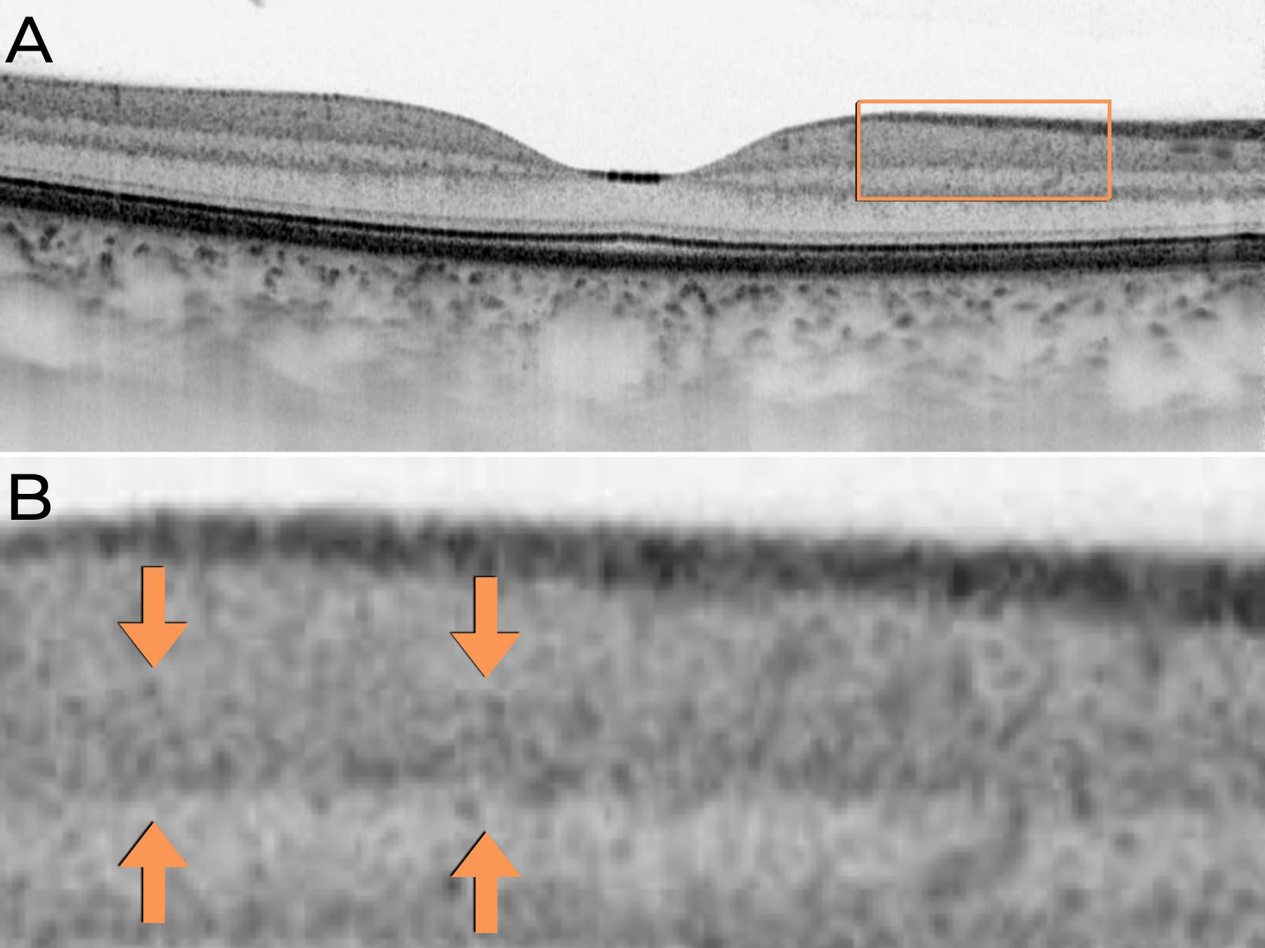
Horizontal macular OCT B-scan acquired using a 6-mm scan length in inverted grayscale mode. (**A**) Inverted grayscale OCT image acquired using a 6-mm scan length with the Topcon Triton DRI device. (**B**) Magnified view of the region outlined by the orange rectangle in (**A**). Orange arrows indicate the IPL region where differentiation of the five strata (IPL 1–IPL 5) is not distinguishable

Subject 1 underwent examinations using the Nidek RS3000 Advance (Fig. 1) and Zeiss Cirrus 6000 (Fig. 2). Subject 2 was examined with the Nidek RS3000 Advance (Fig. 3), while Subject 3 underwent imaging with the Topcon Triton DRI device (Fig. 4). The technical specifications of the devices, along with the acquisition parameters used for all scans, are summarized in Table 1.

**Table 1:** Technical specifications of the optical coherence tomography devices

Parameter	RS-3000 Advance	CIRRUS 6000	Triton DRI
Scan protocol	Line	Line HD 100x*	Line
A-Scans per B-Scan	1024	Not specified*	1024
B-Scan Repetitions	120	100*	128
Axial resolution (optical)	7 μm	5 μm	8 μm
Axial resolution (digital)	3 μm	1,95 μm	2.6 μm
Resolution (Transverse/Lateral)	20 μm	12 μm	15 μm
Maximum Scanning Speed (A-scans/sec)	53.000	100.000	100.000
OCT technology	Spectral domain	Spectral domain	Swept source
Wavelength (nm)	880	840	1,050
A-Scan Depth	2.1 mm	2.9 mm	2.6 mm
Noise Reduction	Yes	Yes	Yes
Eye Tracking	Yes	Yes	Yes

*The device has fixed presets that do not allow modifications

The B-scan images were analyzed using the device’s proprietary software, which allows toggling between standard grayscale and inverted grayscale visualizations. No external software or digital image manipulation was applied to analyze any of the images.

In **Case****1**, the B-scan image obtained using the traditional grayscale mode did not allow clear differentiation of the strata within the IPL, as shown in Fig. 1A. However, when the grayscale was inverted, partial outlines of hyper-reflective lines composing the IPL became discernible (Figs. 1B and 2). In **Case 2**, the B-scan images covering a 3-mm area, while maintaining the same A-scan density, allowed clear visualization of the multilaminar pattern and precise delineation of the boundaries of the five strata within the IPL in the parafoveal region. This clarity was consistently observed in both horizontal and vertical scans (Fig. 3), and the bands were slightly less reliable than other inner retinal bands.

In **Case 3**, the B-scan images covering a 6-mm area (the smallest configuration allowed by the device), while maintaining the same A-scan density, did not allow for the distinction of the five strata of the IPL (Fig. 4).

In cases 1 and 2, the visualization of the pentalaminar pattern of the IPL exhibited regional variations corresponding to the physiological regional differences in IPL thickness. Adjacent to the fovea, where the IPL is thinner, differentiation of the distinct strata within the IPL was not possible. Conversely, in the parafoveal region, where the IPL is thicker, the pentalaminar pattern was easily visualized. This pattern remained consistent throughout the perifoveal region.

Additionally, some hyperreflective dots were observed above the IPL-1 sub-band, situated very close to this layer in certain regions and exhibiting slightly increased reflectivity compared to the IPL sublayers, along with some degree of posterior shadowing. These findings align with the sectional view of capillaries forming the superficial capillary plexus of the retina.

## Discussion

These findings challenge the long-standing notion that the stratification of IPL cannot be visualized using conventional SD-OCT. We demonstrate herein that a multilaminar pattern resembling IPL sublayering can be qualitatively appreciated using standard, commercially available technology, without experimental hardware or post-processing. While our results do not rival the anatomical detail achievable with HR-OCT or VIS-OCT, they suggest that optimized imaging protocols and grayscale inversion can reveal consistent reflectivity gradients aligned with the expected IPL structure, particularly in the parafoveal region.

The ability of OCT to resolve structures at an intracellular level has been increasingly explored in clinical practice, enabling the identification of objective biomarkers widely utilized for functional assessment, treatment planning, and prognosis [3]. While conventional SD-OCT can distinguish 11 different retinal “layers,” recent studies using experimental devices have enabled the analysis of up to 28 bands, including the five strata within the IPL (referred to as IPL-1 to IPL-5) [3]. While the identification of IPL sub-bands is achievable, the experimental nature of these devices remains a substantial barrier to their widespread use in clinical studies [2, 3].

### SD-OCT image acquisition

While image manipulation is a common practice in other medical specialties, such as radiology, the ophthalmologic literature lacks comprehensive information on the advantages and disadvantages of different SD-OCT capture methods and B-scan display formats [4]. Conventional SD-OCT devices allow customization of the scan dimensions, A-scan density, and B-scan repeatability. Additionally, they feature specific software for eye tracking and image optimization [5].

Although the performance of SD-OCT devices is often highlighted by the industry in terms of their A-scan acquisition speed, other factors, such as scan density and repeatability, should also be considered [5]. For instance, the quality of the final B-scan image depends on A-scan density and the number of repeated B-scans in the same region, which together contribute to generating the optimized image [5]. Additionally, the precision of eye-tracking systems, which ensure that rescans target the exact same retinal region, is crucial for image quality [5]. These systems prevent the inclusion of B-scans captured at different positions during image processing, thereby avoiding artifacts [5]. Examination duration, in turn, is influenced by the device’s A-scan acquisition speed, the number of B-scans performed, and the efficiency of the eye-tracking system [5].

Importantly, the axial resolution of SD-OCT (~ 5–7 μm) surpasses the estimated thickness of individual IPL sublayers (~ 2–4 μm), inherently limiting anatomical resolution. As such, the stratification observed in our study likely represents reflectivity gradients or transitions between overlapping signal layers, rather than discrete anatomical boundaries. Additionally, assessment was based on subjective visual interpretation, with no quantitative analysis or formal interobserver agreement. Grayscale inversion may have introduced visual artifacts. Such limitations must be considered when interpreting IPL visualization using standard SD-OCT technology. No quantitative metrics, such as contrast analysis, were applied in this study, which represents a limitation and a direction for future work.

Commercially available devices are typically configured with default capture parameters optimized for shorter examination times, enabling greater productivity and generating smaller digital file sizes to conserve storage space [5]. However, these configurations often involve reducing A-scan density and B-scan repeatability, which can negatively impact image resolution and quality [5]. This often-overlooked factor is significant and should be considered when evaluating the capability of conventional SD-OCT to identify retinal structures. In the present study, we utilized scans with high A-scan density (1024) and high B-scan repeatability (120) for 3-mm and 12-mm line scans. The smaller scan dimension (3 mm) was deemed superior for identifying IPL sub-layers.

### SD-OCT image display

Once acquired, the information about retinal structure obtained through SD-OCT can be displayed in various formats, including color, grayscale, and inverted grayscale (also referred to as “negative display”) [4, 6]. While color-scale images are visually appealing, they can be misleading due to the arbitrary assignment of colors to reflectivity values, as determined by the instrument manufacturer [6]. The human eye’s heightened sensitivity to color differences may create the illusion of significant changes where only minor variations in reflectivity exist, potentially leading to misinterpretations of the underlying tissue structure [6].

Although the human eye can distinguish millions of color variations, it can differentiate only a few hundred levels of grayscale [6]. Paradoxically, this limitation enhances the ability of grayscale images to accurately present OCT data by reducing artificial color transitions [6]. Grayscale OCT images outperform color images in visualizing subtle details and pathologies, such as the integrity of the photoreceptor layer and the morphology of epiretinal membranes [6]. This superiority arises from grayscale’s avoidance of the artificial transitions inherent in color scales, allowing for a more precise depiction of reflectivity changes [6].

Despite evidence supporting the superiority of grayscale over color for SD-OCT interpretation, limited research has explored the potential advantages of grayscale and inverted grayscale in visualizing specific retinal structures and conditions [4, 6]. Although the inverted grayscale display format does not alter the original information, evidence suggests that distinct morphological features are more effectively visualized using this method [4]. The present study corroborates these findings, providing a novel demonstration of this method’s ability to identify all five IPL strata. Notably, this result was achieved without applying image manipulation, such as brightness or contrast adjustments, underscoring the potential for further discoveries through additional techniques.

SS-OCT operates at a longer wavelength (~ 1050 nm), which enhances choroidal penetration but reduces axial resolution and contrast for inner retinal structures. This occurs because longer wavelengths scatter less within retinal tissue, leading to lower spatial discrimination of fine structural details like IPL sublayers. In contrast, SD-OCT uses shorter wavelengths that provide higher axial resolution and better delineation of subtle intraretinal reflectivity diferences, making them more suitable for imaging inner retinal layers.

### Functional and structural insights into the IPL

**Fig. 5:**
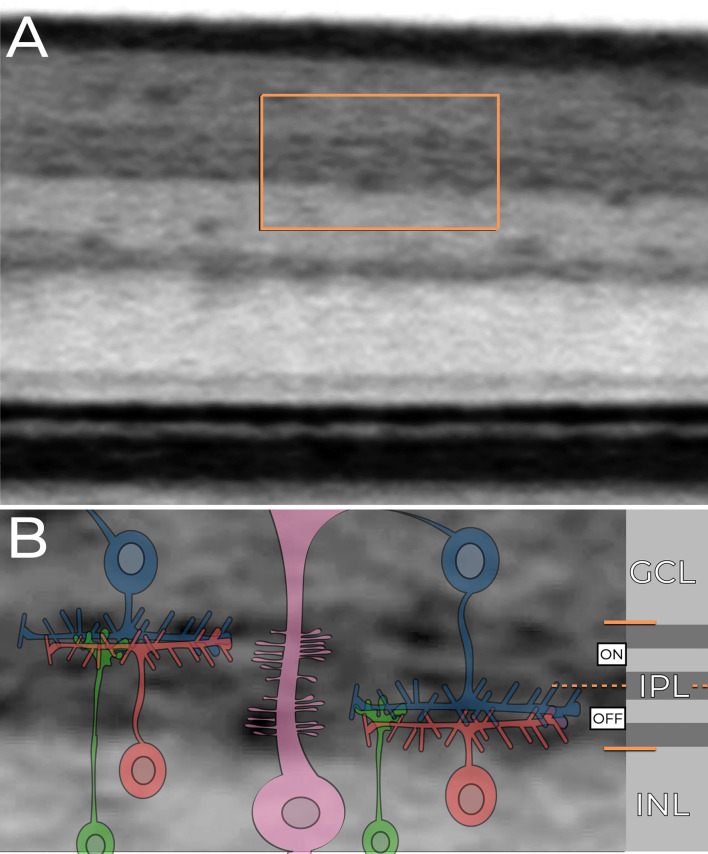
Retinal Cellular Organization in the Parafoveal Area Demonstrated by SD-OCT and Schematic Overlay. The B-scan spectral-domain optical coherence tomography (SD-OCT) image of the parafoveal region, displayed in an inverted grayscale format (top panel), highlights a specific area delineated by an orange rectangle. This region is magnified in the bottom panel, where a detailed schematic overlay illustrates the retinal cellular organization across distinct layers. The highlighted region includes the ganglion cell layer (GCL), the inner plexiform layer (IPL), and the inner nuclear layer (INL). The schematic overlay in the bottom panel visualizes the ON and OFF systems of the IPL, with retinal neurons color-coded by cell type for clarity. The GCL is composed of ganglion cell bodies (dark blue), which form synapses within strata 1 and 2 of the IPL (ON system) and strata 4 and 5 (OFF system). The IPL also contains processes of amacrine cells (red), bipolar cells (green), and Müller cells (pink), which contribute to maintaining synaptic architecture. Adjacent to the image, a grayscale depiction of retinal layers and boundaries corresponds directly to the SD-OCT reflectance data, providing anatomical and functional context. The INL accommodates the cell bodies of amacrine cells, bipolar cells, Müller cells, and other retinal structures, integral to the retinal microarchitecture

The IPL is a complex synaptic network in the retina responsible for processing visual signals from photoreceptors and transmitting them to ganglion cells, which relay this information to the brain [1]. Each of the five IPL sub-bands (S1–S5) contains specific synapses that contribute to the intricate neuronal activity underlying visual perception [1]. Bipolar cells transmit signals from photoreceptors to ganglion cells, while amacrine cells provide lateral inhibitory input, refining signal flow and shaping receptive fields [2, 7]. This interplay of excitation and inhibition defines the response properties of ganglion cells, enabling the retina to extract visual features such as contrast, motion, and color [2, 7]. Fig. 5 presents a schematic representation of the complex cellular organization of the IPL overlaid on the structural aspects observed in SD-OCT imaging.

The location of synapses within IPL sub-bands reflects the functional specialization of distinct cell types [1, 2]. These sub-bands correspond to the segregation of ON and OFF visual pathways, which encode light increments and decrements, respectively [1, 2]. The outermost strata (S4 and S5) represent the OFF pathway, receiving input from OFF bipolar cells [2, 3]. Conversely, the innermost strata (S1 and S2) constitute the ON pathway, where ON bipolar cells transmit signals triggered by light increments [2, 3]. The middle stratum (S3) serves as a transitional zone, integrating inputs from both ON and OFF bipolar cells [2, 3].

Qualitative and regional variations in IPL sub-layers observed in this study closely align with findings from HR-OCT studies [3]. When comparing the illustrative images from HR-OCT studies with those obtained using SD-OCT in the present study, we observed that the IPL demonstrates significant similarities between the two methods [3]. However, this similarity was not noted with VIS-OCT technology, which produces images of superior definition, allowing for more precise analysis and delineation of these bands [2]. Among other technical factors, this difference may be attributed to the greater scattering of shorter wavelengths used in VIS-OCT, particularly within the inner retinal synaptic layers, compared to the longer wavelengths employed in SD-OCT and HR-OCT [1]. While HR- and VIS-OCT offer superior resolution, they are not clinically available. Our SD-OCT approach, using scan optimization and grayscale inversion, allows partial IPL stratification in routine settings.

### Clinical and research implications

Although the precise role of the IPL in major retinal diseases remains incompletely understood, its importance extends beyond ophthalmology—particularly into neurology, where it plays a critical role in evaluating neurodegenerative diseases [11, 12]. The use of SD-OCT in studying these conditions has gained prominence, especially in Alzheimer’s and Parkinson’s diseases, where a well-established association exists between reduced IPL thickness and these disorders [11, 12]. Clinically, improved IPL imaging holds the potential to facilitate the early identification of subtle retinal changes associated with such diseases.

While our findings are preliminary and primarily research-oriented, they suggest that standard SD-OCT may contribute to the detection of subtle inner retinal changes, pending validation in larger, functionally correlated studies. In parallel, this approach provides a foundation for investigating the organization of retinal circuits and their alterations in disease, enabling non-invasive, in vivo structural analyses to support biomarker discovery and treatment evaluation.

## Conclusions

The findings of this study represent a significant milestone in IPL research, showcasing access to valuable information previously deemed unattainable using widely available SD-OCT devices. While SD-OCT enables detailed IPL visualization under specific conditions, SS-OCT does not appear to be well-suited for this purpose.

These results lay the groundwork for future research aimed at refining imaging protocols, establishing normative datasets, and evaluating the clinical relevance of IPL stratification in both ophthalmologic and neurodegenerative conditions. Further studies should focus on validating these findings across larger cohorts, assessing reproducibility, and quantifying their diagnostic and prognostic implications in retinal pathology.

## Data Availability

No datasets were generated or analysed during the current study.

## Declarations

### Generative AI and AI-assisted technologies in the writing process

During the preparation of this work the author(s) used ChatGPT in order to improve language and readability. After using this tool/service, the authors reviewed and edited the content as needed and take full responsibility for the content of the publication.
